# Using Ultrasound and Power-Assisted Devices through Lateral Incision: The OCCULT Technique for Gynecomastia—A Multicentric Large Series Study

**DOI:** 10.1055/s-0045-1802557

**Published:** 2025-02-24

**Authors:** Rajat Gupta, Priya Bansal, Anmol Chugh, Saumil Girish Shah, Gautam Chaudhary

**Affiliations:** 1 Department of Plastic and Reconstructive Surgery, Excel Hospital, C K Birla Hospital, Rosewalk Hospital, New Delhi, India; 2 Department of Plastic and Reconstructive Surgery, CK Birla Hospital, Gurugram, Haryana, India; 3 Plastic Surgery Division, Department of Plastic and Reconstructive Surgery, Skinzone Aesthetics, Borivali, Mumbai, India

**Keywords:** gynecomastia, power-assisted liposuction, ultrasound-assisted liposuction, pull-through technique, surgical outcomes

## Abstract

**Introduction:**

Gynecomastia is a common condition characterized by male breast enlargement, which can have a profound psychological impact on affected individuals. Surgical intervention is often sought to alleviate these concerns. This study evaluates the lateral port technique for gynecomastia surgery, which combines power-assisted liposuction (PAL) and ultrasound-assisted liposuction (UAL) to achieve optimal surgical outcomes with no scar on front of chest.

**Materials and Methods:**

A retrospective analysis was conducted on 967 patients who underwent gynecomastia surgery using the Out of Sight, No Cut on Front of Chest Using Ultrasound and Power-Assisted Devices through Lateral Incision (OCCULT) technique between January 2022 and December 2023. The procedures were performed at multiple centers located in New Delhi, Gurugram, and Mumbai, India. This technique involves a single lateral chest wall incision and incorporates both PAL and UAL to ensure effective tissue removal and contouring. Outcomes assessed included surgical efficacy, complication rates, and patient satisfaction. The surgeries were performed by a team of experienced surgeons, ensuring the reproducibility and consistency of the method.

**Results:**

The OCCULT technique demonstrated high efficacy and high patient satisfaction. Among the 967 patients, 93.3% reported being satisfied with the surgical results. Excellent scar quality was reported by 97.1% of the patients. Moreover, the scar was out of sight, that is, no scar on the front of chest was made in the OCCULT technique for gynecomastia surgery. Complications were minimal, with only 0.8% experiencing seroma formation and no cases of necrosis observed.

**Conclusion:**

The OCCULT technique for gynecomastia surgery is an effective and reproducible method that provides aesthetically favorable results with minimal complications. Its ability to address the psychological and physical burdens of gynecomastia highlights its value in modern surgical practice.

## Introduction

Gynecomastia, derived from the Greek words *gynos* (woman) and *mastia* (breast), is a benign condition characterized by increased glandular and fatty tissue in the male chest. 1 Its incidence ranges from 4 to 69% in adolescents, with 36% persisting into adulthood. 2 This condition can lead to significant psychological concerns, including anxiety, social phobias, and depression. 3 Common causes include increased estrogen levels, decreased androgen levels, and idiopathic factors. 1 4 In adult males with long-standing gynecomastia, medical treatments are often ineffective, leading to surgery as the preferred option due to the fibrous nature of the tissue. 4 5

The surgical goal is to achieve a flat, contoured chest with minimal visible scars, enhancing patient confidence with or without clothing. Early techniques involved surgical excision of fat and gland, often leaving scars. One of the reasons for patients coming for gynecomastia surgery is they avoid going bare chest for swimming/beach vacation. If there are visible scars on the front of the chest after surgery, then complete rehabilitation of the patient is not achieved.

In 1983, Teimourian and Perlman introduced liposuction for gynecomastia, yet incisions near the nipple areola complex (NAC) were still necessary, leading to complications such as visible scarring and altered sensation. 5 Morselli later introduced the “pull-through” technique with two small incisions, but it struggled with the removal of dense glandular tissue. 4 Subsequent advancements like ultrasound-assisted liposuction (UAL) and power-assisted liposuction (PAL) improved outcomes. 6 7 8

This article presents a refined technique combining PAL and UAL for effective removal of dense fibroglandular tissue through a single small incision, strategically placed on the lateral chest wall. This approach minimizes visible scarring by employing liposuction (using the SAFE [separation, aspiration, and fat equalization] technique) 9 and the pull-through method for gland removal, 10 via a single lateral incision. Simon's classification for gynecomastia was used, with grades I, IIa, and IIb treated effectively using this technique. 6

## Materials and Methods

This retrospective cohort study included 967 patients treated consecutively between January 2022 and December 2023. The study was a multicentric study conducted across multiple centers in New Delhi, Gurugram, and Mumbai, India. Approved by the GeneBandhu: Independent Ethics Committee (Ref- ECG031/2024), the study excluded cases of pseudo-gynecomastia and grade III gynecomastia.

Preoperatively, patients were marked in a standing position to delineate areas for liposuction and incision sites. Standardized preoperative photographs were taken. All surgeries were performed as daycare procedures under general or local anesthesia with sedation, based on patient preference. Patients were positioned supine with the arms abducted.

The procedure comprised five steps:

1. *Infiltration and separation:* Infiltration was performed with a 4-mm basket cannula connected to a power-assisted device through an infiltration pump, delivering a solution of 1-L normal saline, 10 mL of 2% lignocaine, and 1 mL of epinephrine (1:1,000). Approximately 300 to 800 mL of fluid was infiltrated per side, facilitating the breakdown of dense fibroglandular adhesions. The role of infiltration is to facilitate the breakdown of fibrous-glandular adhesions. During infiltration, a power-assisted system with a basket cannula is used, which simultaneously aids in breaking these adhesions.
2. *Fat emulsification with Vibration Amplification of Sound Energy at Resonance (VASER)* : VASER was utilized to emulsify fat in superficial and deep planes, aiding in gland loosening for the pull-through technique and promoting skin contraction. Apart from this, UAL also creates an air brush effect at the tip of the probe. This reduces the need for cross-tunneling.
3. *PAL:* PAL was performed with straight and curved 4-mm basket cannulas through a single lateral chest wall incision. In tougher fibrous cases, 5-mm cannulas were used. The average aspirated fat volume per side ranged from 350 to 950 mL. The 4-mm basket cannula (with a basket area diameter of approximately 6–7 mm) is introduced through a 4-mm incision by angling the cannula and sliding it inside the incision. The incision does not need to be larger than the flared dimension of the cannula.
4. *Gland excision (pull-through technique)* : Glandular tissue was removed using Allis forceps through the lateral incision, ensuring precision. In the cases where gland tissue resisted removal, small sections were removed piecemeal, excising with a no. 11 blade through the same lateral incision only. The no. 11 blade is not introduced into the 4-mm incision. Instead, the gland is pulled out using a pair of Allis forceps, and if necessary, it is gradually incised under direct vision.
5. *Fat equalization:* Fat equalization was performed using a 4-mm basket cannula to smooth out irregularities. The incision was typically closed with a single 5–0 Ethilon suture, and a closed suction drain was applied in cases of significant lipoaspirate. The drain is placed through the same incision using a 16/18 drain in selected cases. It is fixed with a suture and removed once the drainage reduces to less than 25 mL/d. The maximum duration for drain placement is 48 hours.

The data collected included patient demographics, gynecomastia grade, and postoperative outcomes such as complications, satisfaction, and cosmetic results. Follow-ups were conducted at 48 hours, 1 week, 3 weeks, 6 weeks, and 6 months.

## Results

Of the 967 patients studied, all presented with bilateral gynecomastia and the mean age was 26.3 years (range: 12–55 years). Most patients (95%) were aged 18 to 35 years, with psychological distress commonly driving the decision for surgery ( Table 1 ).

**Table 1:** Demographic profile

Parameters	No. of patients ( *n* = 967)	Percent
Age group (y)		
12–17	11	1.1
18–30	725	75.0
31–40	216	22.3
41–50	12	1.2
>50	3	0.3
Mean ± SD (range)	26.3 ± 5.7 (12–57)	

Abbreviation: SD, standard deviation.

Most patients had grade IIa (78.2%) gynecomastia, followed by grade IIb (15.4%) and grade I (6.4%) gynecomastia ( Table 2 ).

**Table 2:** Grade of gynecomastia

Grade of gynecomastia	No. of patients ( *n* = 967)	Percent
I	62	6.4
II	756	78.2
III	149	15.4

Note: Simon's classification of gynecomastia.

Almost all patients (99.3%) underwent liposuction with gland removal (UAL + PAL). Only seven patients (0.7%) required additional periareolar gland excision due to dense tissue ( Table 3 ).

**Table 3:** Type of surgery

Type of surgery	No. of patients ( *n* = 967)	Percent
Liposuction (UAL + PAL) + gland removal	960	99.3
Liposuction (UAL + PAL) + periareolar gland excision	7	0.7

Abbreviations: PAL, power-assisted liposuction; UAL, ultrasound-assisted liposuction.

Fluid infiltration during surgery ranged from 300 to 800 mL (average: 450 mL), fat aspiration from 350 to 950 mL (mean: 549 mL), and gland resection from 20 to 125 g (average: 56 g). Surgery duration averaged between 45 and 90 minutes.

**Fig. 1:**
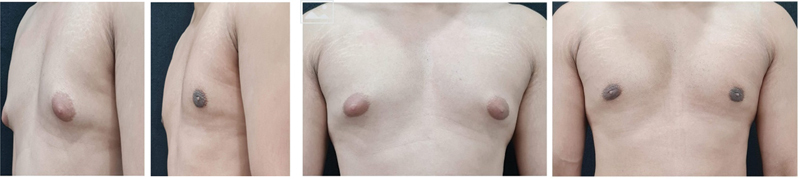
Before and after pictures a 32-year-old patient with grade II gynecomastia with 375 mL of lipoaspirate on each side.

**Fig. 2:**
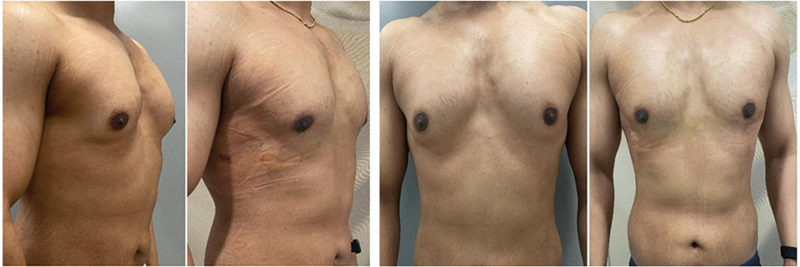
Before and after pictures of a 22-year-old patient with grade II gynecomastia with 250 mL of lipoaspirate on each side.

**Fig. 3:**
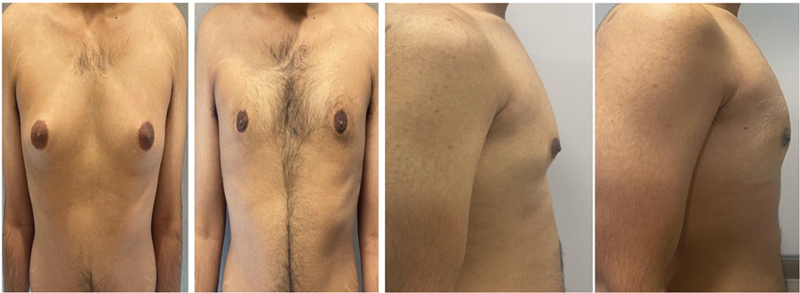
Before and after pictures of a 26-year-old patient with grade II gynecomastia with 335 mL of lipoaspirate on each side.

**Fig. 4:**
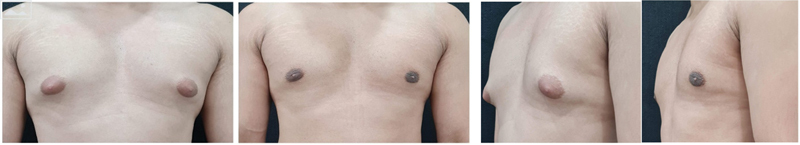
Before and after pictures of a 29-year-old patient with grade II gynecomastia with 300 mL of lipoaspirate on each side.

Complications included seroma in 0.8%, crater formation in 0.5%, and hematoma in 0.2%. No cases of NAC necrosis, infection, or dehiscence were reported ( Table 4 ). Patients were highly satisfied at the 2-week follow-up (93.3% were highly satisfied); 96.7% reported excellent breast contour, 97.1% reported excellent scar quality, and 96.3% reported no significant discomfort or pain ( Table 5 ). Figs. 1 and 2 represent the side and front views of patients who underwent gynecomastia surgery. Fig. 3 shows a close-up view of the scar 6 months postsurgery. Follow-ups revealed that only seven patients (<1%) required a periareolar incision due to dense tissue, particularly in those with a history of anabolic steroid use ( Figs. 4 5 6 7 ).

**Table 4:** Postoperative complications reported

Complications	No. of patients ( *n* = 967)	Percent
Seroma	8	0.8
Hematoma	2	0.2
NAC necrosis	0	0.0
Induration	39	4.0
Crater/saucer deformity	5	0.5

Abbreviation: NAC, nipple areola complex.

**Table 5:** Quality of surgical outcomes (shape, scar quality, and pain)

Quality of surgical outcomes	No. of patients ( *n* = 967)	Percent
**Shape**		
Excellent	935	96.7
Good	32	3.3
Fair	0	0.0
Poor	0	0.0
**Scar quality**		
Excellent	939	97.1
Good	28	2.9
Fair	0	0.0
Poor	0	0.0
**Discomfort: pain**		
No	931	96.3
No response	36	3.7

**Fig. 5:**
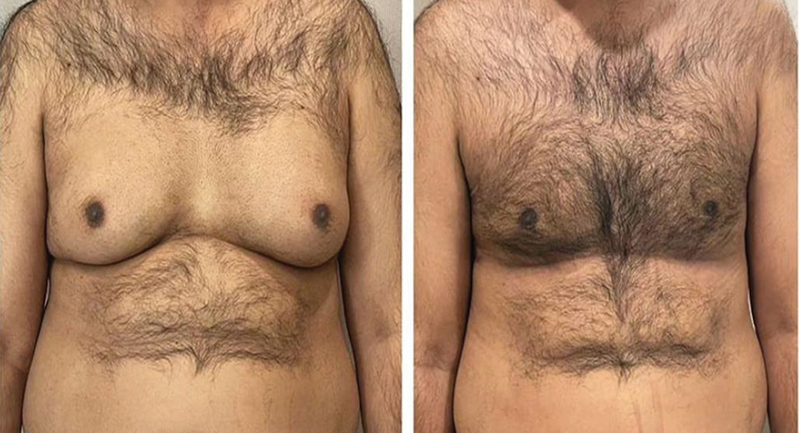
Before and after pictures of a 38-year-old patient with grade III gynecomastia with 750 mL of lipoaspirate on each side.

**Fig. 6:**
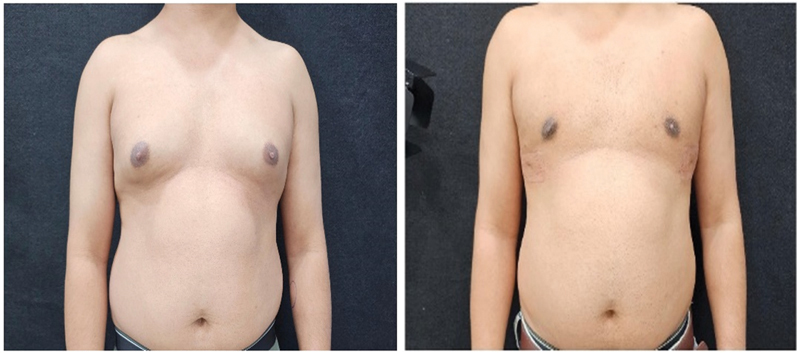
Before after pictures of a 42-year-old patient with grade III gynecomastia with 585 mL of lipoaspirate on each side.

**Fig. 7:**
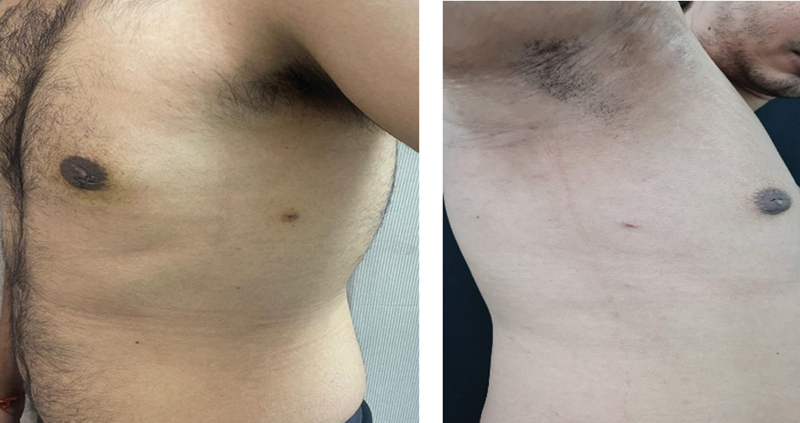
Close-up view of scar 6 months postsurgery.

Minor complications included mild induration in 4% of patients, treated with triamcinolone acetonide injections. Saucer deformity occurred in 0.5% of cases, with one requiring fat grafting. Seromas developed in 0.8 of patients with grade IIb gynecomastia, resolving with conservative management. Importantly, no hematoma, NAC necrosis, or infections were noted, reinforcing the approach's safety. Mild bruising resolved within 3 weeks.

## Discussion

Gynecomastia surgery has undergone significant evolution over recent years, with particular focus on optimizing patient outcomes and minimizing visible scarring. Early gynecomastia surgeries primarily involved the removal of glandular tissue via submammary or periareolar incisions, often resulting in visible scarring. However, the introduction of the pull-through technique by Morselli 11 represented a breakthrough in minimizing scarring while effectively excising glandular tissue. This technique, aimed at providing better aesthetic results, has now been integrated with advanced liposuction modalities to further enhance the outcomes of gynecomastia correction.

A notable advancement in liposuction technology has been the development of UAL and PAL. The application of UAL in gynecomastia treatment has been shown to improve the precision of fat removal while promoting skin retraction. 2 Studies have demonstrated that UAL promotes tighter skin, especially in patients with higher-grade gynecomastia or significant fatty tissue. 6 Similarly, PAL provides a more efficient method for fat removal, reducing the risks of irregular contouring, and enhancing overall results. 5 7 These technologies have become essential tools in gynecomastia correction, allowing for improved outcomes, especially in complex cases where both glandular and fatty tissues must be addressed simultaneously. 2 8

Moreover, combining the pull-through technique with UAL and PAL has proven to be highly effective in achieving optimal chest contour, providing both glandular excision and fat reduction with minimal scarring. 5 6 UAL, with its skin-tightening properties, has been particularly advantageous in patients with significant skin redundancy, as it aids in the retraction of skin postsurgery. 9 This synergy between liposuction and gland excision offers enhanced results, resulting in a more masculine chest contour and reducing visible signs of surgery, which is a critical factor in patient satisfaction. 2 6

However, despite the advantages, there are certain limitations associated with this combined approach. As highlighted by Sattler and Gout, 12 the learning curve for using these advanced liposuction technologies is steep, and surgeons unfamiliar with these tools may experience longer operative times during their initial stages of adoption. Additionally, some patients with excessive glandular tissue or significant skin laxity may require additional incisions, such as a periareolar incision, to achieve the desired outcome. 3 For patients with more severe cases, staged procedures may be necessary to allow natural skin retraction, as outlined by Morselli and Morellini, 13 who observed that such approaches have been particularly effective in treating high-grade gynecomastia with significant skin redundancy.

Despite these challenges, the combined approach has significantly reduced complications, such as hematomas, infections, and contour irregularities. A multicenter review by Lista et al 14 highlighted that using PAL and the pull-through technique together has minimized the incidence of hematomas and wound dehiscence, contributing to faster recovery and lower revision rates. Moreover, these methods have helped achieve a smoother chest contour, which is essential for both aesthetic outcomes and patient satisfaction. 7

In the cases where the tissue is dense or fibrous, such as in patients with more severe forms of gynecomastia, there may be challenges in complete tissue removal through the small lateral incision. However, combining UAL with the pull-through technique has been shown to enhance tissue removal efficiency in such cases as well. 6 In addition, emerging technologies like radiofrequency-assisted lipolysis may complement UAL in achieving more uniform results in patients with less elastic skin. 5

Another important aspect of modern gynecomastia surgery is the consideration of the psychological impact on patients. Studies have consistently shown that improved chest contour can significantly enhance a patient's quality of life by reducing body image concerns and increasing confidence. 9 Patients who undergo successful surgery report high levels of satisfaction, especially when the aesthetic results align with their expectations of a more masculine chest. 2 Furthermore, as noted by Hidalgo and Elliot, 15 the evolving paradigm of gynecomastia management focuses not only on physical correction but also on improving patient experience, which has been a key component of this advanced approach to surgery.

Several patients after traditional periareolar approach though still remain conscious of their scars (being on the front of the chest) when they are bare chested. This precludes their complete rehabilitation in social life and patients still avoid going for swimming or on beach vacation. The OCCULT technique of gynecomastia also solves this issue, ensuring that the scar is out of sight on the lateral chest wall and not on the front of the chest, making patients acceptability and satisfaction higher after this surgical technique. In fact, the location of scar is such that bilateral scars can never be visible together to anyone else from any view. Hence, this also avoids any social stigma later on for the patient and there is no visible sign of having gynecomastia surgery in their past.

Although challenges such as skin management and steep learning curve remain, the integration of UAL, PAL, and the pull-through technique represents a significant leap forward in the treatment of gynecomastia. As the field continues to advance, the incorporation of 3D imaging for preoperative planning and precision, as well as the exploration of additional complementary technologies, may further optimize outcomes in future practices. 14

The OCCULT technique, a combination of minimally invasive techniques such as PAL, UAL, and the pull-through method for gland excision, provides exceptional results in the treatment of gynecomastia. These techniques have led to a reduction in complications, enhanced patient satisfaction, and a more refined aesthetic outcome. As research in this field progresses, we can anticipate further innovations that will continue to improve the safety, efficacy, and overall patient experience of gynecomastia correction. 16

## Conclusion

This innovative technique combines PAL, UAL, and a lateral pull-through approach to enhance the safety and effectiveness of gynecomastia surgery. With minimal scarring and superior cosmetic outcomes, it is a versatile, reproducible method that has been proven to be safe across a large patient population.
